# Intestinal Hæmorrhage in an Infant

**Published:** 1875-07

**Authors:** Norman Bridge

**Affiliations:** Chicago


ARTICLE III.

### A CASE OF INTESTINAL HÆMORRHAGE IN AN INFANT ONE DAY OLD.

BY NORMAN BRIDGE, M.D., CHICAGO.

Mrs. J. T. W., a healthy American woman of 30 years, had borne two healthy children, the youngest being two years old. She gave birth, May 1st, 1875, at 10 A. M., to an apparently healthy girl, at full term, and after an easy labor. The child was to all appearance perfectly normal. It slept most of the time until the next morning at 3 o'clock, when it nursed for the first time. It nursed three times within an hour and went to sleep again. It did not waken for more than a moment until 10.30 A. M., when it was found to have had a dejection of almost pure venous blood. The dejection was large, and was followed within half an hour by two others of the same material, each one successively being less copious. With each discharge from the bowels there was vomiting of dark grumous matter, small in quantity. There was little or no outcry, and the child died at 2 o'clock, of exhaustion.

The autopsy (twenty-four hours after death) revealed the descending colon enormously distended, it being in some places one and one-half inches in diameter. It was greatly elongated ; the loop of the sigmoid plexure reaching across the abdomen to the right iliac region. This part of the intestine was likewise deeply congested, dark red in color, and very much thickened. The mucous membrane was rough, softish, and covered with a dirty mucus. The gut contained chiefly gas and clots of blood. The whole of the large intestine was more or less congested, but the small intestine appeared normal. The stomach was enlarged ; its walls were thickened and congested, and its cavity was filled with a black mixture, like the matter vomited, only darker in color.

The heart, liver and lungs were normal.

It ought to be stated, however, that at birth the child was covered with a large amount of offensively dirty slime, and the fluid forced out of the womb immediately after the expulsion of the child had this appearance of offensiveness so markedly, that at the time it was suggested that meconium had been discharged before birth.

There was nothing learned in the family history that could throw any light on the cause of the phenomena observed in this case.

